# Serious games and eating behaviors: A systematic review of the last 5 years (2018–2022)

**DOI:** 10.3389/fnut.2022.978793

**Published:** 2022-09-08

**Authors:** Pierpaolo Limone, Giovanni Messina, Giusi Antonia Toto

**Affiliations:** Learning Science Hub, Department of Clinical and Experimental Medicine, University of Foggia, Foggia, Italy

**Keywords:** eating behavior, serious games, review—systematic, unhealthy, game for health

## Abstract

**Background:**

Serious game intervention has emerged over the years as a popular strategy for solving the problem of unhealthy eating behavior. This has prompted several scholars to explore its significant impact on eating behaviors, identifying its positive effect on nutritional knowledge and eating behaviors. However, since this research field is yet nascent, an update in knowledge is required to further inform the real-world practice as an alternative intervention for instating healthy eating behavior. Therefore, this current research utilized a systematic review method to reveal the latest state of this concept of a serious game and eating behavior, to identify the position of the literature and shed light on under-researched and emerging areas by recommending future investigations.

**Method:**

To achieve the object of this research, four electronic databases- Science Direct, Web of Science (WoS), APA PsyclNFO, and Emerald- were searched using predefined keywords (search string) relating to the review topic. A total of 15,107 results were retrieved from the databases. After title, abstract, and full-text screening, 15 studies were included following inclusion criteria.

**Key findings:**

The result of this research demonstrated that various designs of serious games comprise an effective intervention for changing eating behavior in both children and adults and addressed the risks of childhood obesity and overweight. The findings also show that the design of the games is co-designed by different specialists such as a nutritionist, psychologist and developer, among others, as either single or multiple players. The effectiveness of the games was attributed to behavior techniques (BT), cognitive theories (CT), and socio-cognitive theories (SCT) of behavior change technique (BCT), incorporating an element of implicit learning in serious games. Feedback and reward were the most reported influencing strategies and self-reporting the evaluation approach.

**Conclusion:**

This research contributed significantly to the body of knowledge in the field of serious games as the most recent review of evidence in the research area. Evidence from 93.33% of the included studies confirmed the effectiveness of serious games in addressing eating behavior. This study concludes that serious games are an effective intervention for improving healthy eating behavior and decreasing unhealthy eating behavior and that various elements of behavior change techniques are essential components of implicit nutritional learning through the games. In addition, it is concluded that the risk of childhood obesity and overweight can be reduced or prevented by leveraging the strength of these games. The need for future research in this field was also pointed out by this study.

## Introduction

### The negative effects of unhealthy eating behaviors

Obesity is now a disease developed all over the world. The World Health Organization (WHO) has defined obesity as a pathological clinical condition that manifests itself with the increase and excess of fat mass both at the subcutaneous and visceral levels, and is equally defined as a multifactorial disease, given by a combination of several factors, including excessive intake of unhealthy foods, reduced physical activity, altered microbiome, congenital alterations, genetic susceptibility, and epigenetic alterations (1). In recent years, this condition has reached quite high levels. A phenomenon in strong growth after the COVID-19 pandemic, as the introduction of blockades and other containment measures has significantly changed the lifestyle and eating behavior of citizens (2). The difficulty in following a balanced diet, the increased consumption of “comfort food” able to reduce stress by increasing the production of serotonin which has a positive effect on mood and reducing physical activity with a consequent increase in sedentary behavior, led to an average weight gain of 4 kg per citizen, mostly in women (about 24% compared to 22% of men) and with a prevalence in the under 30s of 21% compared to the general population (3).

For healthy growth and development, the importance of eating behavior has received significant attention over the years. This is due to the increasing incidence of chronic disease and illness that result from unhealthy eating behavior (4, 5). Several studies have shown that unhealthy eating behavior, especially during young age, is a significant risk factor in causing long-term chronic disease. This pattern of unhealthy eating behavior negates the standard eating behavior, consequently increasing the risk of excess weight gain and obesity (6, 7). This may be why Lobstein et al. (8) and Williams et al. (9) rued the drastic increase in the child obesity rate in the past decades and emphasized the necessity for proper guidance for healthy eating behavior.

A Mexican study found that 35.6% of children of the school-age are obese due to unhealthy eating behavior, and as a result may suffer severe emotional and physical health issues (10). To prevent the negative consequences, Nishtar et al. (11) suggested that the intake of healthy food must be encouraged, and unhealthy eating behavior discouraged, especially the consumption of unsaturated fatty foods, which are consumed by over 70% of the people. Ogden et al. (12) argued that while several factors have led to the increase in the rate of obesity, eating behavior has contributed significantly. The increased incidence of obesity due to unhealthy eating behavior has been reported not only in Mexico but across regions. Instituto Brasileiro de Geografia e Estatística (13) research also revealed an exponential growth in the rate of overweight people in Brazil, again attributing it to diet decisions. Another research found that Brazilian adolescent consumption of all kinds of saturated fats and sugar exceeds the standard recommendation for consumption level, falling short of the recommended intake of other essential minerals needed for growth and development (14). Mendes (15) point out that addressing these challenging public issues is quite difficult because several factors such as social, cultural, etc. predict eating behaviors.

### The role of serious games intervention in reducing unhealthy eating behavior

According to literature, the most effective interventions for reducing unhealthy eating behaviors are represented by those interventions that foster a non-sedentary lifestyle and healthy eating habits by improving health quality, reducing weight, and preventing weight gain (16). Specifically, among them, we can find diet and lifestyle interventions (17), drugs for weight reduction (18), balancing prescription of medications that cause weight gain (18) and surgical intervention (19). Among these interventions, research has shown that serious games could influence people to be more active, promoting non-sedentary lifestyles (20).

Serious games can be defined as games designed for other purpose that pure entertainment (21) and the emergence of serious games intervention has become popular over the years in solving the problem of unhealthy eating behavior. Baranowski et al. (22) describe the serious games intervention as an emerging and complementary intervention approach to accomplish that need through exciting, innovative, and enticing approaches for luring attention, enlightening and enhancing attitudes and human behavior change. Long before Baranoskwi and Shrum (23) described the games as a “rule-based system” with different outcomes depending on the individual performance of the players. Earlier, the author indicated that due to this rule-based system, players must overcome the physical or mental challenges posed along the line of the games to accomplish the goal of the game, and as such become emotionally connected to the outcome.

Unlike the aforementioned interventions used to reduce and prevent obesity, this approach has been established to identify unhealthy eating behavior and train people in healthy eating behaviors through exciting and innovative attraction, without preforming the player. These games are interactive mechanisms that encourage a flexible association with the content, and as a result, the players become familiar with the learning process. Rabin (24) and Schell (25) described long back that the main contribution of this game lies in the players' understanding of the focus, process of design, and impact of the game. Emphasis was placed on how interactive the game is with the player.

In addition, several authors have discussed the positive effect of serious games on nutritional knowledge and eating behaviors (22). Jonhson-Glenberg and Heckler (26) using the Video game Allien health intervention on children, revealed that children who underwent intervention with this game had increased understanding of nutritional knowledge and good eating behavior, vis-a-vis the standard of MyPlate guideline by the US Department of Agriculture, compared to other children. A similar and recent study by Hermans et al. (27) revealed that playing the game for just an hour increases children's knowledge of the most important macronutrients of food. Playing Fit, Food, and Fun Video games by young children was reported by Holzmann et al. (28) to increase children's nutritional knowledge of food intake. Likewise, Matchetti et al. (29) confirmed, with a sample of children above 14 playing videos, the other scholars' position regarding increased nutritional knowledge resulting from the game (30).

Some other scholars describe the game as an educational tool for changing eating behaviors. The educational learning approach is based on implicit and explicit strategies. According to DeSmet et al. (31), it uses explicit educational strategies, e.g., provision of an answer, feedback, and suggestion to the game players, while the implicit learning strategies educate the player with prior awareness (32). The significant attention attracted by this field is proportional to the increase in the number of mobile games and serious games for health and educational purposes. Likewise, its potential benefits in terms of stimulating players' behavior account for its increasing popularity. This game is reported to stimulate players' brain function through the improvement of their behavior and cognitive performance (33). Several scholars have posited that serious games bring about positive effects. Charlier et al. (34) found serious games to be an effective and positive intervention for enhancing children's healthy eating behavior. Moreover, several scholars have reported positive results of the serious games intervention in healthy eating behavior. Majumdar et al. (35) found that children between 11 and 13 playing the serious video game creature 101 had an increase in nutritional knowledge, which eventually lead to a drastic decrease in the consumption of high-sugar beverages and processed snacks by the children after introducing the intervention. A similar study by Sharwama et al. revealed positive results of video game intervention. The study reported that children playing Quest to Lava Mount serious video games for 6 weeks increased their nutritional behavior and reduced consumption of sugar.

Although research in this area is still scarce, serious games interventions seem to be promising interventions to influence healthy eating habits such as non-sedentary lifestyle or modifications of eating patterns, promoting behavioral changes in users (36).

### Characteristics of effective serious games interventions

The process and design of the games were revealed as a moderator of the player's experience, and as such, the predictor of outcomes. Savi and Ulbricht (37) highlight that for the effectiveness of the game through a more engaging learning experience for the player, the game should be designed in such a way that the gameplay, rules of the game, interactions of the key elements, adaptations, actions, feedback mechanisms, winning, conflicts, resolutions, and understanding in terms of interpretation should all have interaction in the design. This is because these associated parameters will bring about increasing player pleasure from the game, due to interactive experience, which is considered an intrinsic driver of the player for playing serious games. All these unique features are expected to enhance an interactive player's experience. These moments of enjoyment experienced by the players while playing serious games can be channeled into an implicit learning message. This may be why games are regarded as lean-forward media, as opposed to the radio and television traditional approach, regarded as lean-back media (38). Hence, serious games are highly effective in attracting and retaining children's attention, and are therefore a good tool in communication, inducing healthy eating behavior by increasing the players' sensitivity to food intake (39).

### The current study

While much is known about the significance of serious games, there seems to be limited research that has distinctively reviewed findings on how serious games can change eating behaviors, although a recent and similar review has been conducted by Ifeoma (40). The research was on serious games and nutritional behavior. To avoid repeating these, the present research aims to remove the limitation of the study and further update the body of knowledge. The fact that this research focuses more on serious game design and the review of findings between 2015 and 2020 affords it insights into identifying unhealthy eating behavior and moderated solutions resulting from serious games in the last 5 years. The research coverage, though originally up to 2020, was extended to 2022, as it is believed that in the space of 2 years (2020–2022) more findings might have been revealed. Furthermore, Ifemoa's research, as also highlighted in their limitations, did not cover relevant database and do not provide a rigors assessment of the included studies using theoretical frameworks that guide the realization of review studies such as the PRISMA method. *Therefore, the present study aims to provide a systematic review of how serious games can change eating behavior, in the last 5 years, with an emphasis on eating behavior*.

## Method and methodology

Since this research aims to synthesize evidence-based information from existing publications on how serious games impact eating behaviors, it has been decided to proceed with a systematic literature review (SLR) approach. According to literature, indeed, this method is significant in terms of evidence synthesis that will aid decision-making and policymaking in real-time (41–44).

**Figure 1:**
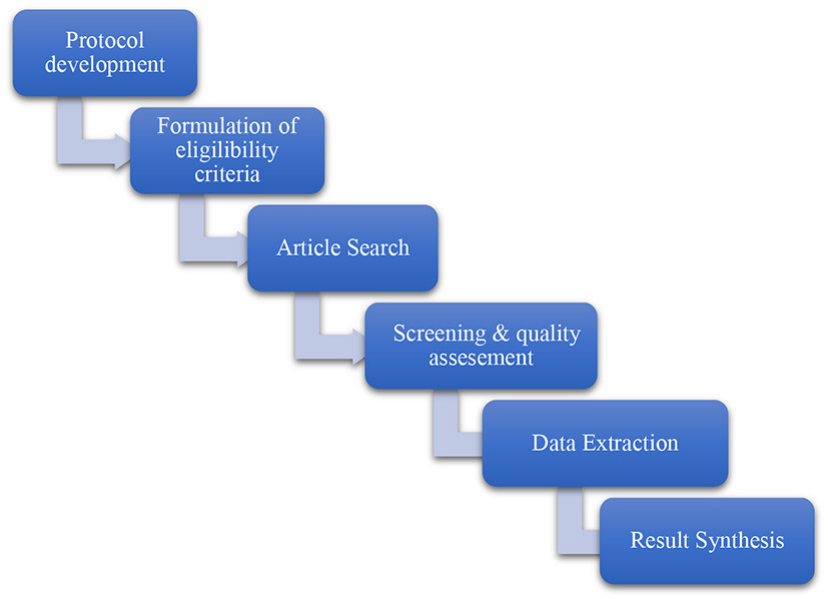
Flow diagram explaining the stages to the identification of relevant studies.

This review utilized qualitative information of secondary data following the guidelines suggested by Petersen et al. (45), to gather and synthetise information from high-quality scientific studies relevant to the topic. The Preferred Reporting Items for Systematic Reviews and Meta-Analyses (PRISMA) protocols were utilized, in incongruence with Moher et al. (46). This helped to manage the identifications of relevant studies as part of the search process, which allowed us to develop eligibility criteria for inclusion and exclusion during the screening process of studies. Only references that met the inclusion criteria were included in the study. The methodology consisted of several stages. The first stage of the study was the protocol development, followed by the inclusion and exclusion criteria. We performed literature research for studies from the database. The studies were screened by two authors. The screening process covered the abstract, title and full-text screening, followed by data extraction and lastly, a synthesis of the previous findings. A flow diagram showing the stages is presented in Figure 1.

### The research protocol

The protocol is the first stage of the research and establishes the main research question that guides the article search, selection of papers, the data sources and search string, the inclusion and exclusion criteria, as well as the results section (47). With the aid of the research questions, quality studies were identified from four different databases.

### Inclusion criteria

The selection process for the inclusion of papers follows the inclusion criteria to detect the overall validity of the literature review; studies were eligible for the research only if they focused on the research topic, which is the impact of a serious game and eating behaviors. Papers were selected from 2018 up to 2022 to retrieve the latest research information in the context of the review topic. Serious games' development processes rapidly change and update. For this reason, this review focus on studies published in the last 5 years to identify the most current trends, in order to present and analyze the most up-to-date interventions. We included papers written in the English language since most of the published peer-reviewed papers are in English and other reviewers too confine their search to English studies to save time and resources and reduce the shortcomings of non-English papers (48, 49). Studies reflecting the search keywords (Serious games and eating behaviors), and original articles were considered as opposed to review papers, for the reliability of synthesis evidence. Likewise, only research fields related to the review topic were considered and articles as the document type were alone included.

### Exclusion criteria

Due to the quality of the current research, we excluded studies not written in English, systematic literature review studies, dissertations, magazines, conference papers, notes, letters, and observational studies. Likewise, publications not falling within the 2018–2022 period were excluded. Since this study concentrated on serious games and eating behaviors, studies that digressed from the scope of the topic and papers were excluded, as also research papers without a clear explanation of serious games.

### Search strategy

The Science Direct, Web of Science (WoS), APA PsyclNFO, and Emerald databases were searched for the publication period of 2018–2022. The appropriateness of these databases is due to their specificity and diversity across domains. For instance, WoS is considered a leading database globally for the search for scientific citations and is diversified across research fields (50). The scope of the literature search was based on the inclusion and exclusion criteria. The database was queried using pre-formed search keywords or strings within the title and abstract of the databases using the Boolean operator (“AND” and “OR”).

The literature search was performed using the following keywords: “*Serious games” AND “eating behaviors” OR “feeding behaviors” OR “Eating habits” OR “feeding habits” OR “Healthy eating” OR “Healthy feeding” OR “Nutrition.”*

**Figure 2:**
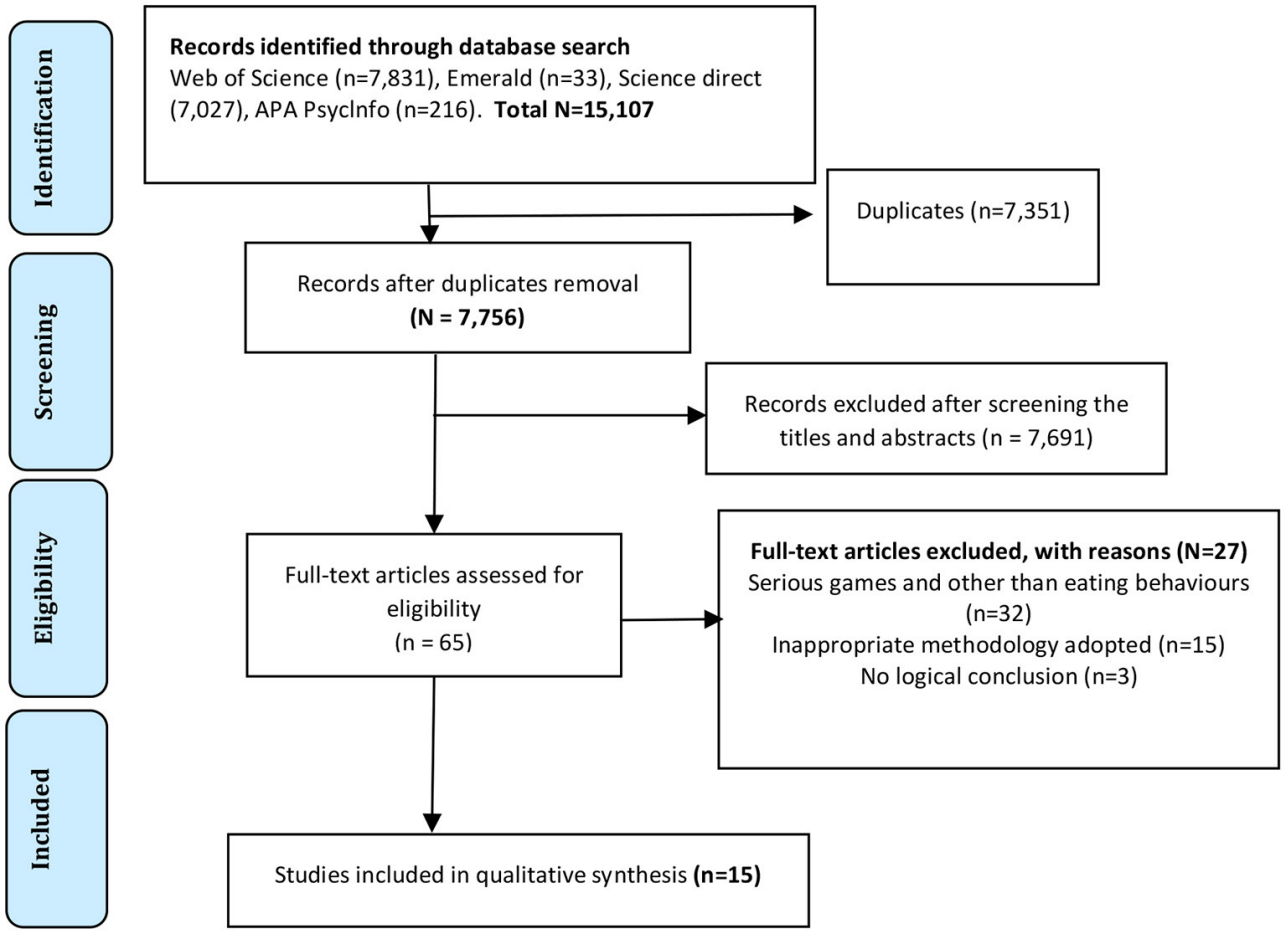
PRISMA flowchart showing the screening process for the identification of relevant studies.

The process of screening using eligibility criteria is shown in the PRISMA flow diagram in Figure 2.

### Study selection and screening process

After applying the keywords to the Science Direct, Web of Science (WoS), APA Psyclnfo, and Emerald databases, 15,107 results were retrieved. The result was then exported into excel software version 12.0. The raw data was cleaned, arranged, sorted and finally, 7,351 duplicates were removed. Then, two independent reviewers scrutinized the title and abstracts of the studies to identify those related to the research, those not within the scope of the paper, and those that did not focus on the serious game and eating behaviors. This led to the omission of 7,691 results.

Differences between the results of the two reviewers were resolved through discussion and engagement. Title and abstract screening were also performed using an excel sheet. Ae full-text screening was performed on the remaining 65 eligible studies to evaluate each study's research questions, method and methodology, data analysis, result presentation, and logical conclusion to see confirm its relationship to the scope of the current study. In this process, 50 papers were excluded because the results of the study were not properly presented, an inappropriate methodology was adopted, no logical conclusions were arrived at, etc. The full-text screening left us with 15 studies to be included in the systematic review. The full-text screening of these was independently performed by the same two reviewers. A consensus was reached on the differences between the two reviewers through discussion. The process of screening using eligibility is shown in the PRISMA flow diagram in Figure 2.

### Quality assessment and data extraction

The quality of the included studies was accessed using the grading of recommendation assessment, development and evaluation (GRADE) by the formulation of certain quality assessment questions and a scoring system of 1–10, similar to the method described by Kitchenham and Stuart (51). The questions are listed in Supplementary Table 1. Each research study accepted as part of the literature was further broken down as per the research topic by applying these questions. Each question represented one point, so each research study had a score ranging from 0 to 10 points. This gave us the opportunity to assess the quality of each included study. In addition, each of the 26 included studies was evaluated based on the formulated questions. The results of the quality assessment are reported in Table 1. The total score across all was rated as “Very Poor” if the total score was <2, “Poor” for scores between 3 and 4, “Good” for scores between 5 and 7, “Very Good” for scores between 8 and 9, and “Excellent” if the total was 10.

**Table 1:** Evidence table.

**A/C**	**Authors, YOP**	**Type of game**	**Description and framework**	**BCT techniques**	**Number of players**	**Game intervention focus**	**Duration**	**Outcome**
S1	Frans et al. (52)	Garfield vs. Hotdog	The game was designed in collaboration with scientific researchers based on a behavior change technique theoretical framework (BCT)	Self-control; Regular and balanced diet; Recognition of healthy and unhealthy foods; Feedbacks on decision	Single	Attitude toward eating, drinks, and snacks	1 week	Not effective
S2	Ismael et al. (53)	FoodRateMaster	Behavioral change theories and techniques were adopted. It included a set of BCTs in the gameplay elements of FoodRateMaster to create a stimulating and engaging environment in which key aspects of healthy behaviors and behavior-specific knowledge were promoted and strengthened It focuses on helping players understand the differences in the nutritional properties of healthy and unhealthy food, as well as the recommended ranges for food nutrients that can help them determine whether they should reduce or maintain the intake of certain foods Behavioral theory (BT), cognitive theory (CT), social cognitive theory (SCT)	BT: Shaping, Behavioral repetition and substitution, stimulus control, learning by consequence e.g., reward CT: Cognitive restructuring SCT: Self-efficacy, self-evaluation, self-observation, and self-reaction	Single	Improve knowledge of healthy and unhealthy foods, increase intake of healthy foods, and reduce intake of ultra-processed food	6 weeks	Effective
S3	Yi-Chin et al. (54)	Fooya vs. Uno	Fooya is a pediatric dietary mobile game with implicit learning components on food choices. It also quantifies children's heterogeneous gameplay patterns using game telemetry and determines the effects of these patterns on players' food choices Uno is a board game without dietary education	Implicit learning; psychoeducation; shaping behavior (modeling)	Single	Choice of food	NR	Effective
S4	Mack et al. (55)	Bursting Bubble Game, Kangaroo-Turtle Race, Liquid Rankings on the Sugar Scale, Foods Under the Microscope, Balloon Game, Relaxation story	Balloon food game: This game deals with the food pyramid and its food groups Food Under Microscope induces satiety, which is the volume of food i.e., dietary energy density (DED) Rankings on the Sugar Scale is a questionnaire scale Kangaroo-Turtle Race: In this game, the player has to apply knowledge about DED without having much time to think. A kangaroo (the computer) races against a turtle (the player) Bursting Bubble Game: This game provides information about eustress, distress, and coping strategies Relaxation Story: This task begins with an introduction about eustress and distress and strategies to cope with stress	Implicit learning; psychoeducation; shaping behavior (modeling)	Single	Influence of KOP on dietary energy density principle (DED-P) concerning nutrition	2 weeks	Effective
S5	Ismael et al. (56)	Helperfriend	Was developed by a multidisciplinary team that included nutritionists, psychologists, physical activity experts, human-computer interaction experts, and software engineers, based on published design methodology. It is a vicarious experiential video game designed to promote 3 lifestyle behaviors among young children: physical activity, healthy eating, and socio-emotional wellness Several BCT were adopted-Behavioral, Cognitive, and Social cognitive theories	Providing information about health consequences, behavioral practice, behavioral substitution, incentives and rewards, goal setting, reviewing behavioral goals, monitoring behaviors, providing feedback on behavior, discrepancies between current behaviors and goals, monitoring emotional consequences, and prompts or cues	NR	Physical activity, healthy eating, and socioemotional wellness	4 weeks	Effective
S6	Chagas et al. (57)	Rango Cards	A digital game developed for an adequate and healthy diet using simple information in a playful context	Implicit learning; psychoeducation; shaping behavior (modeling)	Multiple	Use of digital games to promote healthy dietary practices	NR	Effective
S7	Froome et al. (58)	Foodbot vs. My Salad Shop Bar	Support school children in learning about Canada's Food Guide; however, its impacts on nutrition knowledge	Feedback and monitoring, social support, shaping knowledge, natural consequences, reward, and threat, quizzes and sub-games requiring a user to catch food and sort food	NR	To see whether the digital game improves children's knowledge of Canada's Food Guide	5 days	Effective
S8	Viggiano et al. (59)	Kaledo	This is an educational board game to improve nutritional knowledge and a healthy lifestyle	Implicit learning; psychoeducation; shaping behavior (modeling)	Multiple	To modify dietary behavior	7 days	Effective
S9	Skouw et al. (60)	The Kingdom of Taste	A game which unites fitting motivators, a fitting social situation, and mere exposure to novel or disliked foods through sensory interactions, to encourage food exploration and possibly change eating behavior in families	Implicit learning; psychoeducation; shaping behavior (modeling)	Multiple	To improve food behavior in families	3 weeks	Effective
S10	Alblas et al. (61)	Skyland	A 2D strategic game in which players have to fight an adversary located at a ground level, who is trying to take down floating islands by forcing unhealthy 156 food upon the inhabitants The healthy game is based on the EC paradigm	Implicit learning; psychoeducation; shaping behavior (modeling)	Single	Food choice behavior	NR	Effective
S11	Hermans et al. (27)	Alien Heart Game: Force choice game, Quick sort game, Build a meal game, Ship runner game, Super shopper	A nutritional healthy video game designed to change food choices	Implicit learning; psychoeducation; shaping behavior (modeling)	Single	Short term effectiveness on nutrition and healthy food choices	2 weeks	Effective
S12	Langlet et al. (62)	HTC VIVE VR system (HTC)	The HTC VIVE VR system (HTC) is an immersive VR technology in this study, consists of a headset (connected to a computer) through which the VR environment can be viewed, two hand controllers that enable interaction with the VR environment, and two base stations that enable motion tracking	Implicit learning; psychoeducation; shaping behavior (modeling)	Single	Virtual game on eating disorders	NR	Effective
S13	Rodrigues et al. (63)	VR	This is a virtual reality game that combines the serious game concept for treating eating disorder	Implicit learning; psychoeducation; shaping behavior (modeling)	Single	Eating disorder	1 week	Effective
S14	Weiland et al. (64)	Kid Obesity Game (KOP)	A type of serious game designed to change the nutritional behavior of children and parents	Implicit learning; psychoeducation; shaping behavior (modeling)	Single	Nutritional behavior	2 weeks	Effective
S15	Brown et al. (65)	Foodbot factory	It is an app game that incorporates BCT	Feedback and monitoring, social support, shaping knowledge, natural consequences, and reward and threat	Single	Eating behavioral change	NR	Effective

AC, article code; YOP, year of publication; NR, not reported.

### Data extraction

A pre-defined sheet was used to gather the following information from the included studies: the Author ID, the author's first name, type of study, year of publication, country of publication, sample size, and main findings.

## Results

### Overview of the included studies

Analysis of the last 5 years' publications (2018–2022) shows that the research field experienced increased publication during 2020; since then, the publication rate has shown a downward trend. All the 15 included studies were published across eight countries. Germany and The Netherlands lead in the number of publications, having three papers each. Brazil, Canada, and Mexico had two each, while Denmark, Sweden, and the USA had one publication each. The result of the quality assessment of the 15 studies revealed that five were excellent in quality rating [S1, S2, S4, S9, S11], seven very good [S3, S5, S6, S7, S8, S10, S15], while three studies were good [S12, S13, S14], as shown in Supplementary Table 2.

Different methods were adopted by the studies. Randomized control trial (RCT) and randomized (R) studies were the most prominent methods. Each method was adopted by five studies [RCT: S1, S2, S3, S4, S14; R: S5, S6, S10, S11, S12]. Two of the studies were pilot studies [S8, S9], while the remaining two [S7 and S15] were randomized control pilot and quasi-experimental mixed-methods studies, respectively. The studies adopted different sample sizes, with most of the studies (13 studies) focusing more on children between the ages of 7–15 years while some used parents along with their children as a post-test or feedback for the effectiveness of the serious game intervention on the children. Three studies including a sample composed by the adult population (>18 years old) were found. One study [S10] which included participants aged more than 18 years [S12] with a sample who had a mean age of 37.6, and [S13] for people aged 23–30 years old, as shown in Supplementary Table 3.

None of the studies reported gender bias in the sample population. The studies adopted different serious game types, as the games were differently designed to focus on their research objective, although all the studies reported similar research objectives, viz., eating or nutritional behaviors. This was the reason they are included in this review. All these serious games were designed around foods such as fruits and vegetables, drinks, snacks, whole grains, and varying types of junk foods (Supplementary Table 3). In the game design, 67% or 10 studies adopted a single design of the serious game and 20% or 3 studies adopted a multiplayer game design, while the remaining studies did not report the player design approach. The theoretical framework adopted by the studies uses behavior change techniques (BCT). Varying numbers of days, weeks, and months of playing the games with accustomed minutes of playing time were adopted by the studies. However, 1–2 weeks were the most reported play days. Interestingly, all the included studies confirmed the effectiveness of serious games on eating behaviors except [S1], which found no moderate effect as shown in Table 1.

## Discussion

Evidence from this systematic literature review of the last 5 years on serious games and eating behavior includes fifteen studies, which is not many. However, this is not surprising since this is an emerging research field, but interestingly, the present research identified that field is becoming under-researched in the last 2 years (2021–2022). This is good evidence to draw the attention of scholars to this research area.

### Research outcomes: The positive association between serious games and healthy eating behaviors

Overall, the serious games developed by all the included studies were embedded with various nutritional information, properties, and consequences of healthy and unhealthy foods. The success of players at every stage of the games was developed to depend on either eating or cooking healthy foods. According to the results, indeed, 14 of the 15 included studies, showed positive associations between playing serious games over time and changing in eating behaviors. In this regard, evidence from Ismael et al. (53) confirmed FoodRateMaster, a serious game, as a viable tool for intervening with people, especially 8–10-year-old children, toward healthy eating behaviors. The game was proven to enhance nutritional knowledge and the rate of food intake behavior of the children who play the game, toward healthy eating behavior. The study also confirmed these from the parents of the children after the intervention, as most of them reported positive feedback for the game in terms of reducing their children's positive attitude toward the consumption of unhealthy foods. Also, Mack et al. (55) proved through the development of different serious games, that these games increase the nutritional knowledge of the player, enhancing the positive attitude toward healthy food intake and their coping ability. In the same way, Chagas et al. (57) developed Helperfriend, a serious game based on behavior change techniques (BCT) to moderate physical activities, eating behavior and socio-emotional behavior. In line with previous scholars, their study confirmed the effectiveness of games in improving the dietary knowledge of the player and food intake behavior. Another serious game developed by Yi-Chin et al. (54) confirmed the significant positive effect of a game named Fooya on children's food choices. Similar to previous research, they also posited that players show a negative attitude toward eating unhealthy food after playing the game. The study iterated that the positive outcome observed in the game is due to the implicit learning behavior embedded in it, which tends to increase the players' nutritional knowledge, haven experience the consequence of unhealthy and healthy eating behavior through the game.

Contrasting findings were reported only by one study (52) who failed to confirm previous findings using the Grafield serious game developed to persuade children to eat fruits as opposed to Hotfdog game for energy-dense food. They found no positive attitude toward eating behavior in the children who played the Grafield game and no negative attitude to eating unhealthy energy-dense food. The food intake behavior was not different between those who played the game and non-players i.e., those who played the game did not eat healthier or unhealthier food than those who did not play. The non-effective result of Frans et al. (52) may be related to the design and approach of the game. Or maybe specifically the game playing time.

### The role of game design

According to the result of this review, a central feature that bring together all the analyzed serious games is the game design. In all the studies, the game design incorporated behavior change techniques (BCT). These techniques aim at moderating the psychology of the game players around eating behavior. The techniques were reported to exist in three major frameworks, including behavioral technique (BT), cognitive theory (CT), and social cognitive theory (SCT). Most of the game designs were around these theoretical frameworks. This element of BCT theoretical constructs induced in the game is said to create a stimulating and interactive environment for players, which in turn may have psychological interaction with the players' brains, thus stimulating their behaviors. The construct is centered on self-control, stimulus control, recognition, repetitive behaviors, social support, feedback, monitoring, shaping of nutritional knowledge, and most importantly, learning by consequence i.e., rewards, threats, and consequences of unhealthy eating (66).

With regard to the single-player o multiplayer design of the serious game, instead, with both the format positive outcomes were found. For example, the Kaledo board game developed by Viggiano et al. (59) is a multiple player serious game proven to make players live a healthy lifestyle by instigating healthy eating behavior. Likewise, the Kid Obesity program Game (KOP) and FoodBotFactory are single-player serious games designed by Weiland et al. (64) and Brown et al. (65). Both games demonstrate how serious games maintain high nutritional knowledge in children who play the game. The game was reported to be interactive and full of fun, which was the key reason for its acceptability among children. KOP helps change even parents' nutritional behavior and can reduce the rate of obesity which mostly increases amongst kids due to unhealthy eating and lifestyles. Virtual reality is another version of a serious game observed to be effective for instigating eating behavioral change (67). Rodrigues et al. (63) and Langlet et al. (62) confirmed how the VR serious games version could be used as an effective intervention for treating an eating disorder.

Although the number of articles reviewed in this study is only 15, results from this review could usually inform future serious games interventions for promoting healthy eating behaviors. Results indicated that it is important to take into account different field of disciplines and experts when designing the serious game's contents (education, psychology, nutrition, human-computer interaction, user experience, engineering). Moreover, they highlighted the importance of adapting the interventions' content to individual differences, to create interventions as tailored as possible for users, to improve their efficacy.

### Limitations

Critical issues arise from this review. First of all, no standards in terms of the evaluation of the efficacy of these interventions based on serious games seem to have been reached. For example, while some interventions were evaluated in terms of pre-and post-performance of participants in both intervention and control groups, other interventions compared only participants' performance over time. Secondly, the majority of studies present interventions that use serious games developed with a one-size-fits-all approach instead of tailoring the games taking to account the peculiar differences and characteristics of individuals or groups to increase their efficacy. Furthermore, most of the studies do not include in their research design a long-term evaluation of the intervention efficacy in order to verify the generalization and consolidation of the learned behaviors. Finally, only three studies have been found with the adult population. These studies showed similar findings in terms of efficacy with studies conducted on children and adolescents. Further studies on the adult population are needed to corroborate these similarities.

The findings of this review should also be interpreted in light of the limitations of our own work. The small number of included studies reviewed by this current research is one of the few limitations of this study. However, this is because of the five-year (2018–2022) inclusion criteria to review the latest trend in serious games and eating behavior knowledge in the emerging research area. This study is also limited in its consideration of databases, as some other relevant studies may have been omitted during the search. Although the four databases searched may also overlap with other databases, considering too many databases may predispose the search to excessive unjustifiable duplicates of searched results. Instead of these, this research identifies the need for future research to employ broader search terms to retrieve and review more studies on serious games and eating behavior. Also, this study identifies the need for more research in this area, as the studies have demonstrated limited publications in this research field.

## Conclusion

The result of this review demonstrated the present state of research on serious games and eating behavior. The synthesized evidence confirmed how serious games can significantly change people across different age groups toward healthy eating behavior and actual food intake. Increased nutritional knowledge due to the game helps to maintain a healthy choice of food and as such, increases negative attitudes toward unhealthy food choices such as sugared, junk, and fatty-related foods. The majority of the games developed by the included studies reported successful outcomes and are designed using behavior techniques, cognitive theories, and socio-cognitive theories of behavior change techniques. The games were co-designed by several specialists. The effectiveness of the game was observed in children, adults >18, and parents, with the effectiveness of the playtime varying from 5 days to 6 months; feedbacks and rewards were the most frequently adopted influencing strategies and a self-reporting evaluation approach was confirmed across all studies.

## Data availability statement

The datasets presented in this study can be found in online repositories. The names of the repository/repositories and accession number(s) can be found in the article/Supplementary material.

## Author contributions

GM: introduction. PL: research and discussion. GT: conclusion. All authors contributed to the article and approved the submitted version.

## Conflict of interest

The authors declare that the research was conducted in the absence of any commercial or financial relationships that could be construed as a potential conflict of interest.

## Publisher's note

All claims expressed in this article are solely those of the authors and do not necessarily represent those of their affiliated organizations, or those of the publisher, the editors and the reviewers. Any product that may be evaluated in this article, or claim that may be made by its manufacturer, is not guaranteed or endorsed by the publisher.
