# Supplementary material for: Serious games and eating behaviors: A systematic review of the last 5 years (2018–2022)
Source: Front Nutr. 2022 Sep 8;9:978793. doi: 10.3389/fnut.2022.978793 (PMC9493252; doi:10.3389/fnut.2022.978793)
Supplement: Supplementary file 1 [file Data_Sheet_1.PDF]

Table S1: Quality assessment questions

Number	QA questions
QA 1	Is the study question relevant to the review topic?
QA 2	Does the study use a good sampling method?
QA 3	What type of research question is asked?
QA 4	Is the study design suitable for the research question?
QA 5	Does the study method describe the essential potential source of bias?
QA 6	Does the study execution follow standard protocol?
QA 7	Is the stated hypothesis tested by the study?
QA 8	Does the study access any data analysis?
QA 9	Is the conclusion inferred from the data?
QA 10	Does the study report any conflict of interest?

Table S2. The result of the quality assessment

AC	YOP	Country	Title	Quality Score	Remarks
S1	2021	The Netherlands	The Effect of a Serious Health Game on Children's Eating Behaviour: Cluster-Randomized Controlled Trial	10	Excellent
S2	2020	Mexico	Nutritional Education and Promotion of Healthy Eating Behaviours Among Mexican Children Through Video Games: Design and Pilot Test of Food Rate Master	10	Excellent
S3	2020	USA	Impact of Paediatric Mobile Game Play on Healthy Eating Behaviour: Randomized Controlled Trial	9	Very Good
S4	2020	Germany	The Kids Obesity Prevention Program: Cluster Randomized Controlled Trial to Evaluate a Serious Game for the Prevention and Treatment of Childhood Obesity	10	Excellent
S5	2020	Mexico	HelperFriend, a Serious Game for Promoting Healthy Lifestyle Behaviours in Children: Design and Pilot Study	8	Very Good
**S6**	2018	Brazil	Rango Cards, a digital game designed to promote a healthy diet: a randomized study protocol	8	Very Good
**S7**	2020	Canada	The Effectiveness of the Foodbot Factory Mobile Serious Game on Increasing Nutrition Knowledge in Children	9	Very Good
**S8**	2018	Germany	Healthy lifestyle promotion in primary schools through the board game Kaledo: a pilot cluster randomized trial	8	Very Good
**S9**	2020	Demark	A Serious Game Approach to Improve Food Behaviour in Families—A Pilot Study	10	Excellent
**S10**	2018	The Netherlands	Investigating the impact of a health game on implicit attitudes towards food and food choice behaviour of young adults	9	Very good
**S11**	2018	The Netherlands	Feed the Alien! The Effects of a Nutrition Instruction Game on Children's Nutritional Knowledge and Food Intake	10	Excellent
**S12**	2021	Sweden	Virtual Reality App for Treating Eating Behavior in Eating Disorders: Development and Usability Study	7	Good

**S13**	2019	Brazil	A Virtual Reality Environment Using Concepts of Serious Games and Gamification for the Treatment of Eating Disorders	7	Good
**S14**	2022	Germany	A Serious Game for the Prevention of Obesity in School Children–Impact of Parent's Involvement: A Randomized Controlled Trial	7	Good
**S15**	2020	Canada	Optimizing Child Nutrition Education with the Foodbot Factory Mobile Health App: Formative Evaluation and Analysis	8	Very Good

Note: *AC=article code, YOP=year of publication*

Table S3: Characteristics of included studies

AC	Study type	Sample Size	Type of Food	Type of Game	Main Findings
**S1**	RCT	Children 8-12 years<br>S=157	Fruits/Energy Dense-Food	Garfield vs Hotdog	The results showed that playing a serious health game did not affect the attitude toward fruits, energy-dense snacks or the intake of fruits or less energy-dense snacks. Serious health games are increasingly considered to be a potentially effective intervention when it comes to behaviour change. The results of the study stress the importance of tailoring serious health games to be effective because no effect was found on attitude or eating behaviour.
**S2**	RCT	Children (29 girls, 33 boys) aged 8-10 years (mean age 9, SD 0.8 years)<br>8 yrs=11<br>9 yrs= 35<br>10 yrs=14<br>39 parents<br>S=60	French Fries, Spinach, Guava, Soft drink,	Food Rate Master	Children aged between 8 and 10 years indicated an increased level of nutritional knowledge and their self-reported frequency intake of two healthy foods, and a decreased level in their self-reported intake of 10 unhealthy foods after playing FoodRate Master. In addition, the participants' parents agreed that FoodRate Master positively influenced their children's attitudes toward several healthy eating behaviours. These results support that health games such as FoodRateMaster are viable tools to help young children increase their food knowledge and improve dietary behaviours.
**S3**	RCT	Children aged 10 to 11 years<br>S=104	3 pairs of healthy and unhealthy **Drinks** (water and Nimbooz, a carbonated soft drink)<br>**Savory snacks** (cashews and Lays potato chips)<br>**Sweet snacks** (raisins and a 5 Star chocolate bar)	Fooya VS Uno	Mobile video games embedded with implicit learning components, which is a type of serious game, showed a strong positive impact on children's food choices immediately following the game. Game telemetry captured children's different play patterns and was associated with behavioural outcomes. These results have implications for the design and use of mobile games as an intervention to improve health behaviours, such as the display of unhealthy food facts during gameplay
**S4**	RCT	Age: 9-12 years<br>S=82	Green foods with a DED up to 1.5 kcal/g,<br>yellow foods with a DED of 1.5 to 2.5 kcal/g,<br>red foods with a DED above 2.5 kcal/g.	Bursting Bubble Game, Kangaroo-Turtle Race, Liquid Rankings on the Sugar Scale, Foods Under the Microscope	The Kids Obesity Prevention program using serious games intervention sustainably increased knowledge in the areas of nutrition and stress coping.

				, Balloon Game	
**S5**	Randomised	Age: 8-12 years	Healthy Food: (Fruits, Vegetables)<br>Unhealthy Food: (Candies, sweetened cereals, and sugary drinks)	Helperfriend	HelperFriend appears to be feasible and acceptable for young children. In addition, this game seems to be a viable tool to help improve the knowledge, the intention to conduct healthy behaviours, and the dietary intake of children; however, a well-powered randomized controlled trial is needed to prove the efficacy of HelperFriend.
**S6**	Randomised	S=8	Real food: fruits, water, natural fruit juice, boiled eggs)#<br>meals<br>cooked on the spot,<br>Industrialized food.: cheese, canned sardine,<br>Mega-industrialised food with excess ingredient	Rango Cards	The game was designed as a food and nutrition education tool based on Brazilian dietary guidelines. We believe that *Rango Cards* will provide a comprehensive experience on the topic, improving the students' autonomy, motivation, and pleasure of learning.
**S7**	RCP	Age: 8-10 years<br>S=73	Vegetables and Fruits, Protein Food, and whole-grain food	Foodbot Vs My Salad Shop<br>Bar	Foodbot Factory has the potential to be an effective educational tool to support children in learning about nutrition.
**S8**	Pilot Study	Age: 7-11<br>S=1313<br>Treatment Group 651 children (341 boys and 310 girls. Control group: 356 children (187 boys and 169 girls)	Unhealthy Junk Foods (candies, chocolate, chewy gum,<br>chips, crackers, fruit juice beverages, snacks, and sugary).<br>Healthy Foods (fruit, fresh-squeezed orange<br>juice, vegetable, and yoghurt)	Kaledo	The present results confirm the efficacy of Kaledo in younger students in primary schools, and it can be used as a useful nutritional tool for obesity prevention programs in children.
**S9**	Pilot Study	Age: 13-15 years<br>S=39<br>Treatment=22<br>Control=17	Fruits and Vegetables: Carrot, Banana, Broccoli, Papaya, Prune, Caper Berry	The Kingdom of Taste	The results of the study showed a positive change in food behaviour in all families. Quantitative results mainly showed an effect on food neophobia
**S10**	Randomised	Age:>18<br>S=128	Fruit: (tangerine, apple, 196 pear, and banana)<br>Chocolate snacks: (peanut chocolate bar, milk chocolate, chocolate<br>197 muffin, chocolate brownie, and chocolate snacks	Sky Islands	The study identified tentative support for the use of 33 health games to influence IAs towards food and positively affect food choice 34 behaviour.
**S11**	Randomised	Age: 10-13 years	Nutrient-dense food and energy-dense food	Alien Heart Game:<br>Force choice game,<br>Quick sort game, Build a meal	Alien Health Game has the potential to improve children's nutritional knowledge in the short term, but may not be strong enough to increase nutritional knowledge and actual eating behaviour in the long term

				game, Ship runner game, Super shopper	
**S12**	Randomized	Mean age: 37.5<br>S=24	Meatballs, potatoes, sauce, and lingonberries	HTC VIVE VR system (HTC)	Participants found the app usable and eating disorder personnel were positive regarding its fit with current treatment methods.
**S13**	NR	S=10<br>Age: 23-30 years	Virtual Reality	NR	VR enhances the eating behaviour of the participant.
**S14**	RCT	Age: 9-12 years<br>S=46	Kid Obesity Game (KOP)	NR	We found that the amount of knowledge remained high in children and parents, suggesting that at the cognitive level, knowledge, especially about nutrition and the DED principle was sustainable.
**S15**	Quasi-experimental mixed method	Age:9 -12 years	Drinks, Protein food, Fruits and vegetables, Animal protein foods, Plant protein	Foodbot Factory	Children find Foodbot Factory to be fun and easy to use and can engage in learning about nutrition, thus changing their food behavior

Note: *AC=article code, YOP=year of publication, COP=country of publication, RCT=randomised control trial, RCP=randomised control pilot, NR=not reported*
